# Identification and validation of NETs-related biomarkers in active tuberculosis through bioinformatics analysis and machine learning algorithms

**DOI:** 10.3389/fimmu.2025.1599667

**Published:** 2025-06-18

**Authors:** Shengfang Xia, Qi An, Rui Lin, Yalan Tu, Zhu Chen, Dongmei Wang

**Affiliations:** Department of Science and Education Division, Public Health Clinical Center of Chengdu, Chengdu, Sichuan, China

**Keywords:** active tuberculosis (ATB), latent tuberculosis infection (LTBI), diagnosis, neutrophil extracellular traps (NETs), machine learning

## Abstract

**Introduction:**

Diagnostic delays in tuberculosis (TB) threaten global control efforts, necessitating early detection of active TB (ATB). This study explores neutrophil extracellular traps (NETs) as key mediators of TB immunopathology to identify NETs-related biomarkers for differentiating ATB from latent TB infection (LTBI).

**Methods:**

We analyzed transcriptomic datasets (GSE19491, GSE62525, GSE28623) using differential expression analysis (|log, FC| ≥ 0.585, adj. p < 0.05), immune cell profiling (CIBERSORT), and machine learning (SVM-RFE, LASSO, Random Forest). Regulatory networks and drug-target interactions were predicted using NetworkAnalyst, Tarbase, and DGIdb.

**Results:**

We identified three hub genes (CD274, IRF1, HPSE) showing high diagnostic accuracy (AUC 0.865-0.98, sensitivity/specificity >80%) validated through ROC/precision-recall curves. IRF1 and HPSE correlated with neutrophil infiltration (r > 0.6, p < 0.001), suggesting roles in NETosis. FOXC1, GATA2, and hsa-miR-106a-5p emerged as core regulators, and 46 candidate drugs (e.g., PD-1 inhibitors, heparin) were prioritized for repurposing.

**Discussion:**

CD274, IRF1, and HPSE represent promising NETs-derived diagnostic biomarkers for ATB. Their dual roles in neutrophil-mediated immunity highlight therapeutic potential, though drug predictions require preclinical validation. Future studies should leverage spatial omics and CRISPR screening to elucidate mechanistic pathways.

## Introduction

TB, caused by *Mycobacterium tuberculosis* (MTB), remains one of the major public health challenges globally. Despite the significant progress made in the global fight against TB in recent years, according to the Global Tuberculosis Report 2024 released by the World Health Organization (WHO) (1), there were an estimated 10.8 million new TB cases worldwide in 2023, a slight increase from the 10.7 million cases in 2022. TB is transmitted through the air, and individuals exposed to an environment with MTB approximately 30% risk of developing LTBI, which is an asymptomatic and non - infectious state (2). However, without timely treatment, about 5% - 10% of LTBI patients with normal immune function may progress to ATB, and those with underlying diseases may develop ATB more rapidly (3). Delayed diagnosis of ATB and LTBI not only facilitates disease progression but also increases the risk of person-to-person transmission, posing a substantial threat to global TB control efforts. Early detection of ATB and LTBI is therefore critical for initiating timely treatment and implementing effective public health strategies to mitigate transmission.

The pathogenesis of TB involves a complex interplay between MTB and the host immune system (4, 5). Upon inhalation, MTB are phagocytosed by alveolar macrophages, triggering a cascade of innate and adaptive immune responses. During this process, neutrophils, as the first line of defense of the host immune system, participate in anti-TB defense through phagocytosis, production of reactive oxygen species (ROS), and release of NETs,web-like structures composed of DNA, histones, and antimicrobial proteins (6, 7). Existing studies have shown that neutrophils are significantly enriched in the blood and bronchoalveolar lavage fluid of patients with ATB (8), and these cells demonstrate their anti-mycobacterial ability by phagocytosing MTB (9, 10). However, excessive activation of neutrophils may lead to immunopathological damage. For example, although the excessive release of NETs can capture MTB, it can trigger pulmonary inflammation and tissue damage (11, 12). This dual role of NETs—host protection versus pathological damage—highlights their complex regulatory function in TB pathogenesis. These discrepancies underscore the necessity to systematically analyze NETs-related genes (NRGs) and clarify their roles in TB pathogenesis.

Distinguishing between LTBI and ATB is particularly challenging due to overlapping clinical and immunological features. Current diagnostic methods, such as tuberculin skin tests (TSTs) and interferon-gamma release assays (IGRAs), cannot reliably predict disease progression or differentiate between active and latent infection (13, 14). Genomic profiling studies have identified transcriptional signatures associated with TB progression, but these signatures often lack specificity for NETs-related pathways (15, 16). This knowledge gap underscores the need for novel biomarkers that can accurately discriminate between LTBI and ATB, particularly in high-risk populations such as household contacts of TB patients. The immune microenvironments of LTBI and ATB differ significantly. In LTBI, the immune response is characterized by a balanced Th1/Th17 cytokine profile, which restricts MTB replication without causing tissue damage (17, 18). In contrast, ATB is marked by a hyperinflammatory state dominated by neutrophil infiltration and pro-inflammatory cytokine secretion, leading to granuloma formation and lung destruction (19–22). These divergent immune responses likely involve distinct NRG expression patterns. Identifying these differentially expressed NRGs could provide critical insights into disease progression and enable the development of targeted diagnostic tools.

This study addresses these unmet needs by integrating bioinformatics and machine learning approaches to systematically analyze NRG expression profiles in ATB and LTBI. By leveraging publicly available transcriptomic datasets (GSE19491, GSE62525, GSE28623), we aim to 1) identify NRGs that are differentially expressed between ATB and LTBI; 2) validate their diagnostic potential using machine learning algorithms; 3) characterize their functional roles in immune cell infiltration and signaling pathways; and 4) predict potential therapeutic targets by mapping NRGs to druggable pathways. These findings may provide a theoretical basis for the development of new treatment strategies, especially by targeting the NETs regulatory pathway to intervene in the immune response of TB and ultimately improve the treatment prognosis of patients.

## Materials and methods

### Data collection

Gene expression datasets related to ATB were obtained from the GEO database (https://www.ncbi.nlm.nih.gov/geo/) using the GEOquery R package (version 3.20) (23). NRGs were curated from previously validated studies (24, 25). A total of 123 NRGs were manually extracted and compiled into a reference list (**Supplementary Table S1**).

### Identification of DE-NRGs

Differential expression analysis was performed using the limma package (version 3.20) (26). Genes with |log_2_ (fold change) | ≥ 0.585 (equivalent to a 1.5 - fold change)and adjusted *p*-value < 0.05 (Benjamini-Hochberg correction) were considered significantly differentially expressed. Subsequently, an intersection analysis was performed between the DEGs and NRGs to screen out significantly DE-NRGs. These genes likely contribute to ATB pathogenesis.

### Functional enrichment analysis

Gene Ontology (GO) and Kyoto Encyclopedia of Genes and Genomes (KEGG) pathway enrichment analyses were conducted using the ClusterProfiler package (version 4.6.0) (27). The analysis results were presented in various visualization methods to help uncover the potential mechanisms of these genes in the occurrence and development of ATB.

### Immune cell infiltration analysis

Immune cell infiltration levels were quantified using the CIBERSORT algorithm with the LM22 signature matrix (28). Gene expression data and the LM22 signature file were processed through the CIBERSORT R script to estimate the relative proportions of 22 immune cell subtypes in each sample. To ensure robustness, only samples with a CIBERSORT output *p*-value < 0.05 were retained for downstream analysis.

For correlation analysis between hub genes (CD274, IRF1, HPSE) and immune cell subsets, Spearman’s rank correlation was applied. Statistical significance was defined as *p* < 0.05. Visualization of correlation matrices and gene-immune cell interactions was performed using linkET (version 0.0.7.4) (29) and ggplot2(version 3.5.1) (30), with color gradients representing correlation coefficients and point sizes indicating statistical significance. The final plots integrated immune cell-cell correlations (lower triangle) and gene-immune cell correlations (upper triangle).

### Identification of hub genes using machine learning algorithms

To identify the key genes associated with ATB, we employed three classic machine - learning algorithms, including SVM - RFE, LASSO, and RF. These algorithms were implemented using the R packages e1071 (31, 32), glmnet (33), and randomForest (34). The overlapping genes of the three algorithms were considered as hub genes, and the results were visualized using the Venn package (35).

### Prediction of potential drug targets

Potential drug targets were identified using the Drug-Gene Interaction database (DGIdb) (version 5.0.8) (36). Drug-gene interaction networks were constructed using Cytoscape (version 3.7.2) (35). Aiming to provide new drug targets for the treatment of ATB.

### Construction of TFs-gene and miRNAs-gene regulatory networks

Based on the regulatory roles of TFs and miRNAs, we used NetworkAnalyst 3.0 (37) and the Tarbase database (38) to construct the regulatory networks of TFs-genes and miRNAs-genes, respectively. By analyzing the transcriptional regulation and miRNA regulation relationships of these genes, we can better understand the mechanism of action of NETs - related genes in ATB. All the regulatory networks were visualized using Cytoscape to intuitively display the interactions among these molecules.

## Results

### Identification and functional characterization of DEGs

**Figure 1:**
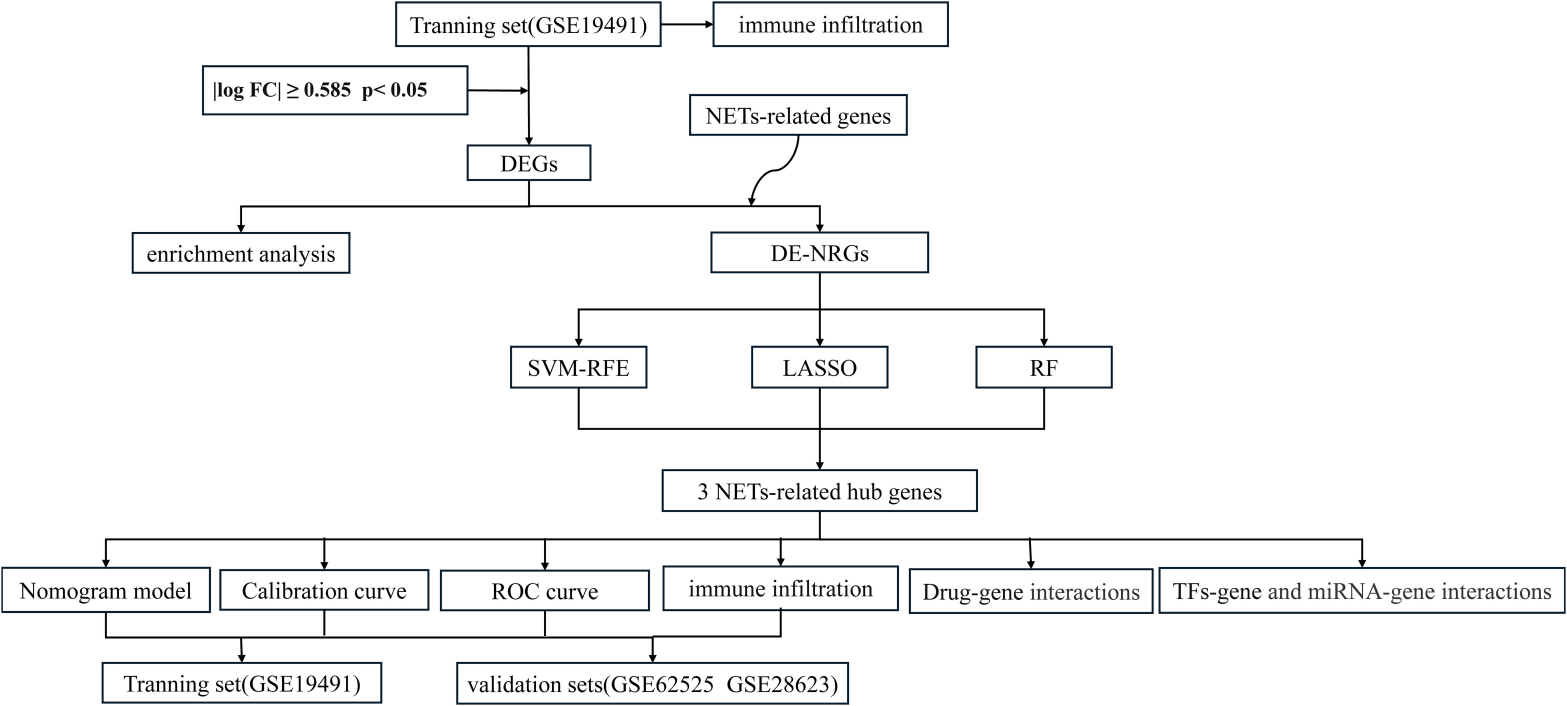
Flowchart of this study.

**Figure 2:**
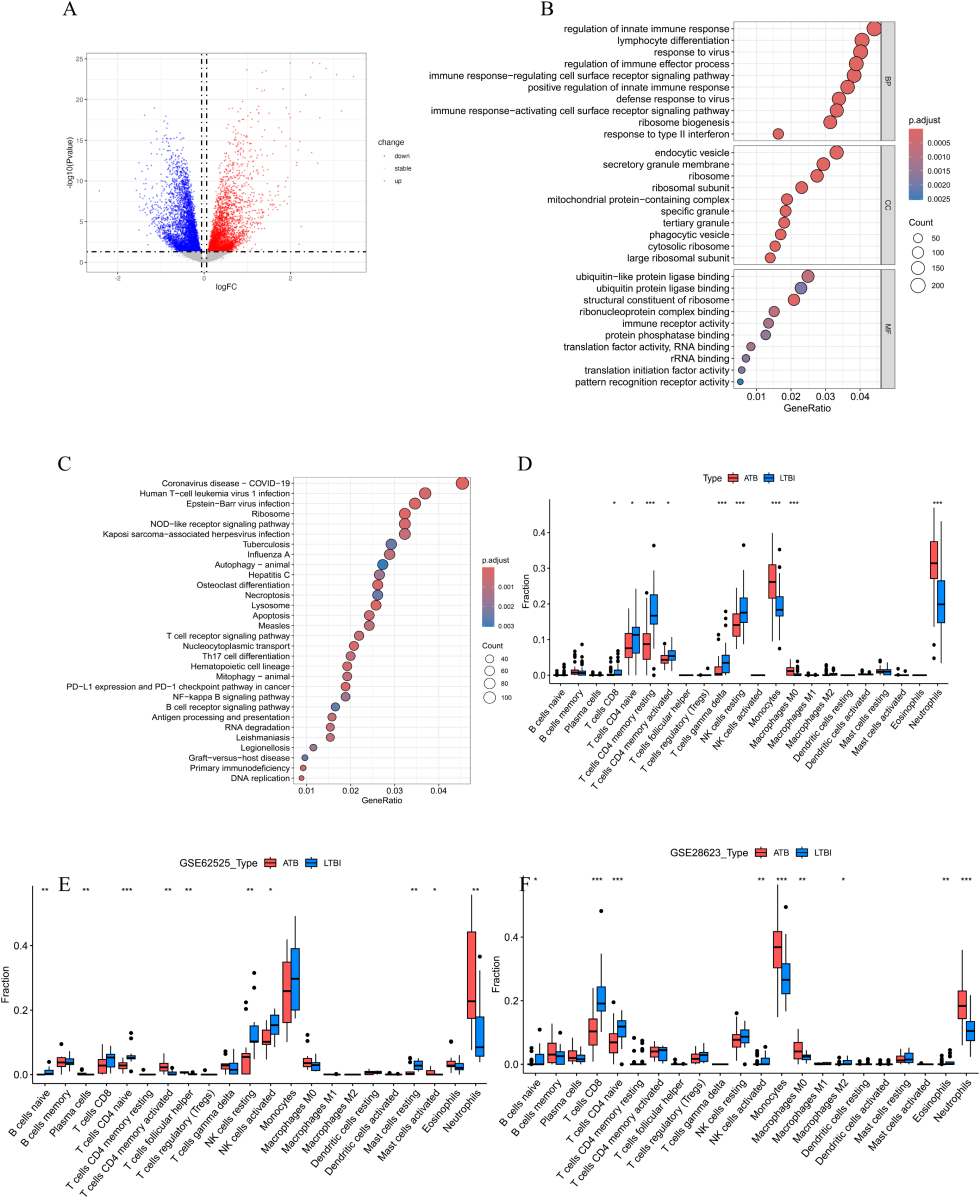
**(A)**The volcano plot of DEGs between ATB and LTBI groups in GSE19491. **(B)** The bubble plots of the GO enrichment analysis results for DEGs, which show Top 10 GO BPs; Top 10 GO CCs; Top 10 GO MFs. **(C)** Bubble plot of KEGG pathway analysis results for DEGs, showing Top 30 KEGG pathways. The size of the bubbles correlates with the number of genes enriched in the pathway, while the color indicates the magnitude of the adjusted p-value, with red indicating a smaller adjusted p-value and blue indicating a larger adjusted p-value. **(D-F)** Comparison of immune cell infiltration profiles between active tuberculosis (ATB) and latent tuberculosis infection (LTBI) patients across three datasets. Relative proportions of 22 immune cell subtypes in ATB and LTBI groups were estimated using the CIBERSORT algorithm in **(D)** GSE19491, **(E)** GSE62525, and **(F)** GSE28623 datasets. Bar plots illustrate the mean infiltration levels of immune cell subtypes (e.g., neutrophils, monocytes, cluster of differentiation 8-positive T cells [CD8^+^ T cells], natural killer [NK] cells, and memory CD4^+^ T cells) in ATB (red) and LTBI (blue). Statistical significance was assessed using the student’s t test (*p < 0.05, **p < 0.01, ***p < 0.001). Error bars represent standard deviation.

The overall flowchart of this study is shown in **Figure 1**. In the GSE19491 dataset, we conducted a differential expression analysis for 69 samples of LTBI and 54 samples of ATB. Finally, a total of 7,959 differentially expressed genes were identified, among which 3,471 genes were upregulated, and 4,488 genes were downregulated in the ATB group (**Figure 2A**). To explore the biological functions and potential roles of these DEGs during the development of ATB, we performed GO and KEGG enrichment analyses. Specifically, a total of 46 significantly enriched KEGG pathways were identified (**Supplementary File 2**), as well as 1,028 GO items (**Supplementary File 3**).

In the GO analysis (**Figure 2B**), we found that in the Biological Process (BP) category, the DEGs were mainly enriched in key immune response pathways such as regulation of innate immune response, lymphocyte differentiation, regulation of immune effector process, and immune response−regulating cell surface receptor signaling pathway. In the Cellular Component (CC) category, the DEGs were significantly enriched in structural components such as endocytic vesicles, secretory granule membrane, and ribosome. In the Molecular Function (MF) category, the DEGs were mainly involved in important functions such as ubiquitin−like protein ligase binding, ubiquitin protein ligase binding, andstructural constituent of ribosome.

The KEGG pathway analysis (**Figure 2C**) indicated that these DEGs were significantly enriched in various pathways, especially those related to immune responses and cell signaling, including the NOD-like receptor signaling pathway, DNA replication, Apoptosis, T cell receptor signaling pathway, Th17 cell differentiation, NF-kappa B signaling pathway, Autophagy–animal, as well as infectious diseases such as Coronavirus disease (COVID-19), Epstein-Barr virus infection, Tuberculosis, etc.

To further explore the role of immune cells in the occurrence of ATB, we used the CIBERSORT algorithm to evaluate the infiltration status of immune cells in the ATB and LTBI groups (**Figure 2D**). According to the results (**Figure 2D**), patients with LTBI exhibited higher levels of CD8^+^ T cells, naïve CD4^+^ T cells, resting memory CD4^+^ T cells, activated memory CD4^+^ T cells, gamma delta T cells, and resting natural killer (NK) cells. In contrast, patients with ATB showed significantly higher levels of monocytes and neutrophils. Analyses in two validation datasets, GSE62525 (**Figure 2E**) and GSE28623 (**Figure 2F**), further confirmed the high infiltration of neutrophils in patients with ATB.

### Identification of NETs-related hub genes in ATB

**Figure 3:**
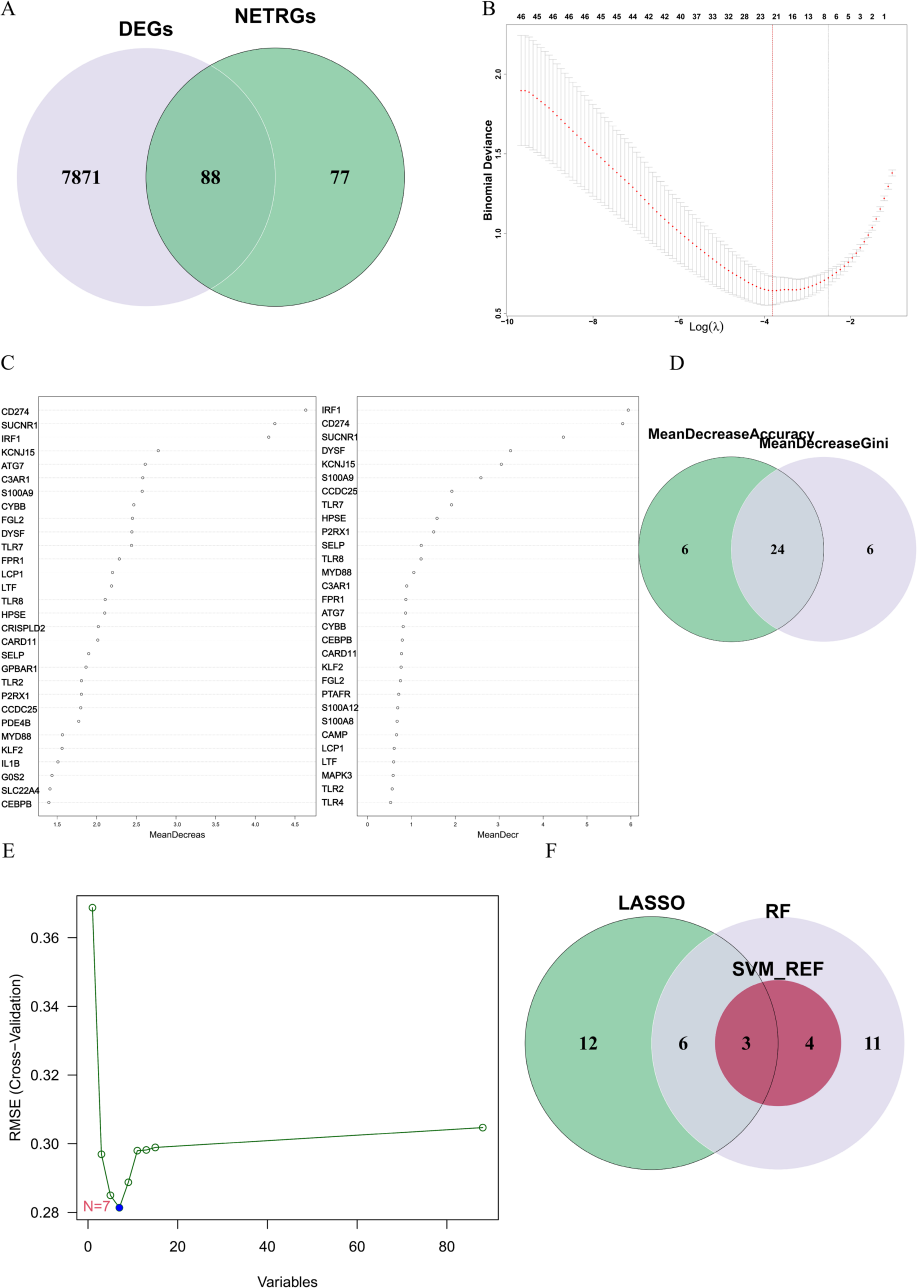
**(A)** The gene overlap between DEGs and NRGs. **(B)** The LASSO algorithm was used to obtain the hub genes associated with ATB, and the error was minimized when 21 genes were included. **(C)** Random forest (RF) algorithm ranked genes by mean decrease accuracy (MDA) and mean decrease Gini (MDG). Top 30 genes from each metric were intersected. **(D)** The overlapping genes between the 30 genes with the mean decrease accuracy and the 30 genes with the mean decrease Gini. **(E)** Error plot of different number of features in SVM-RFE. The minimum error was obtained for the inclusion of 7 genes. **(F)** Venn diagram showing the overlap of candidate genes for the above three machine learning algorithms.

To identify the DE-NRGs closely associated with the occurrence of ATB, we performed an intersection analysis between the DEGs and the known NRGs, obtaining 88 DE-NRGs (**Figure 3A**). Subsequently, we used three machine learning algorithms (SVM-RFE, LASSO, and RF) to screen key genes from these DE-NRGs.

In the LASSO algorithm (**Figure 3B**), with the best lambda of 0.0220116, 21 key genes were identified. In the RF algorithm (**Figure 3C**), by setting the optimal number of trees to 65, we conducted an intersection analysis of the top 30 genes ranked by “mean decrease Accuracy” and “mean decrease Gini”, ultimately screening out 24 key genes (**Figure 3D**). The SVM-RFE algorithm (**Figure 3E**) identified 7 key genes.

Finally, through the intersection of the results from the three algorithms, we identified 3 key hub genes: CD274, IRF1, and HPSE (**Figure 3F**). These genes are considered NETs-related hub genes and may play important roles in the pathogenesis of ATB.

### Identification and validation of the diagnostic value of NETs-related hub genes

**Figure 4:**
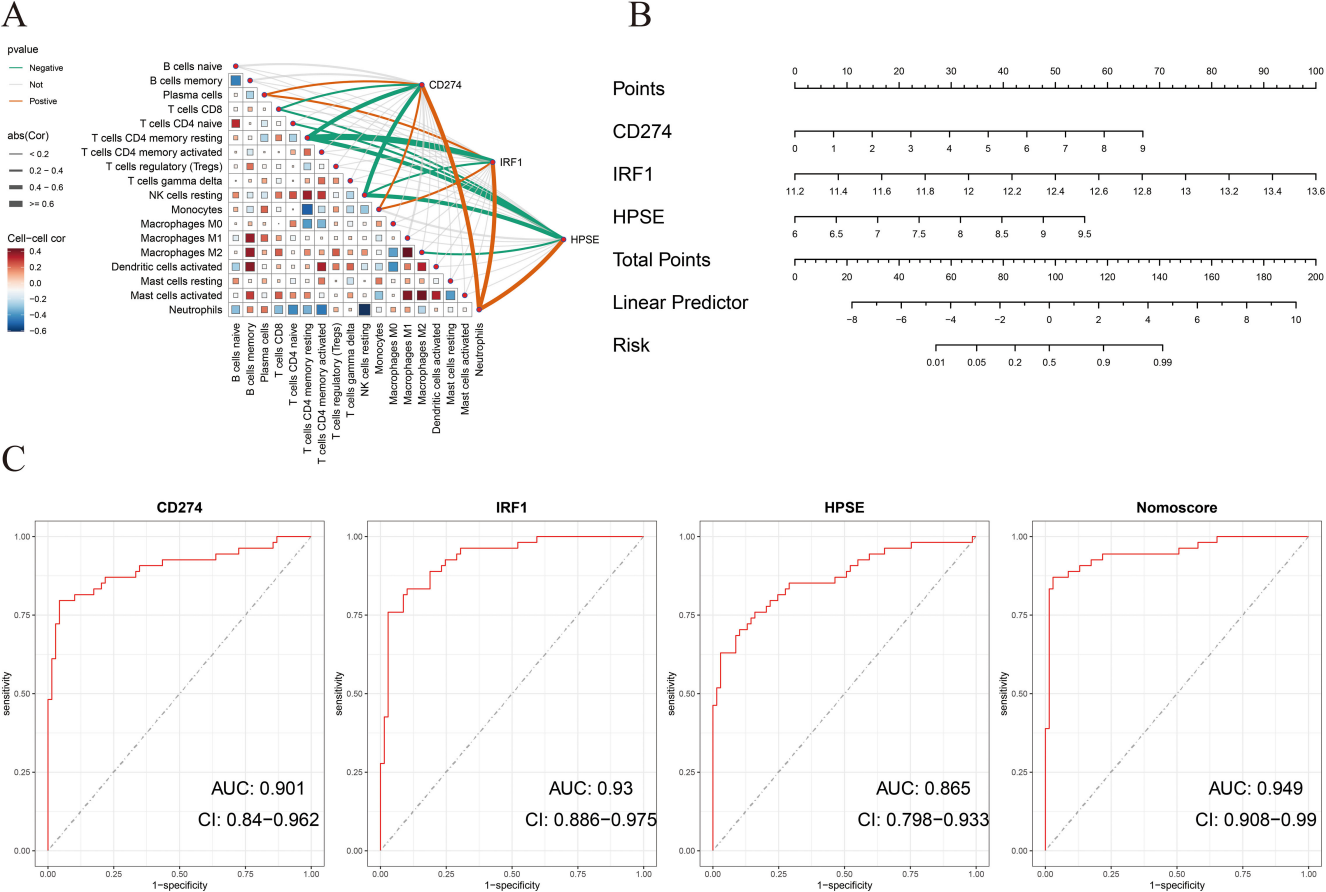
**(A)** Correlation between immune cells and between NETs-related hub genes and immune cells in the GSE19491 dataset. **(B)** Nomogram construction of three NETs-related hub genes in the GSE19491 dataset. **(C)** ROC curve of the three NETs-related hub genes and nomogram in discovery datasets for GSE19491.

To determine the diagnostic value of the above three hub genes in ATB, first, this study analyzed the correlations between the three genes and immune cells. As shown in **Figure 4A**, in the training dataset GSE19491, CD274, IRF1, and HPSE were significantly positively correlated with neutrophils. Then, a nomogram model was constructed based on the three genes. The relative expression level of each gene corresponded to a score, and the total score was calculated by adding up the scores of each gene (**Figure 4B**).

ROC curve analysis indicated that all three genes had good diagnostic performance. **Figure 4C** shows that for CD274 (AUC: 0.901, 95% CI: 0.84 - 0.962), IRF1 (AUC: 0.93, 95% CI: 0.886 - 0.975), HPSE (AUC: 0.865, 95% CI: 0.798 - 0.933), and the Nomoscore (AUC: 0.949, 95% CI: 0.908 - 0.99). Surprisingly, it could be inferred from the AUC values that CD274 and IRF1 had outstanding diagnostic efficiency, while HPSE also had good diagnostic value.

**Figure 5:**
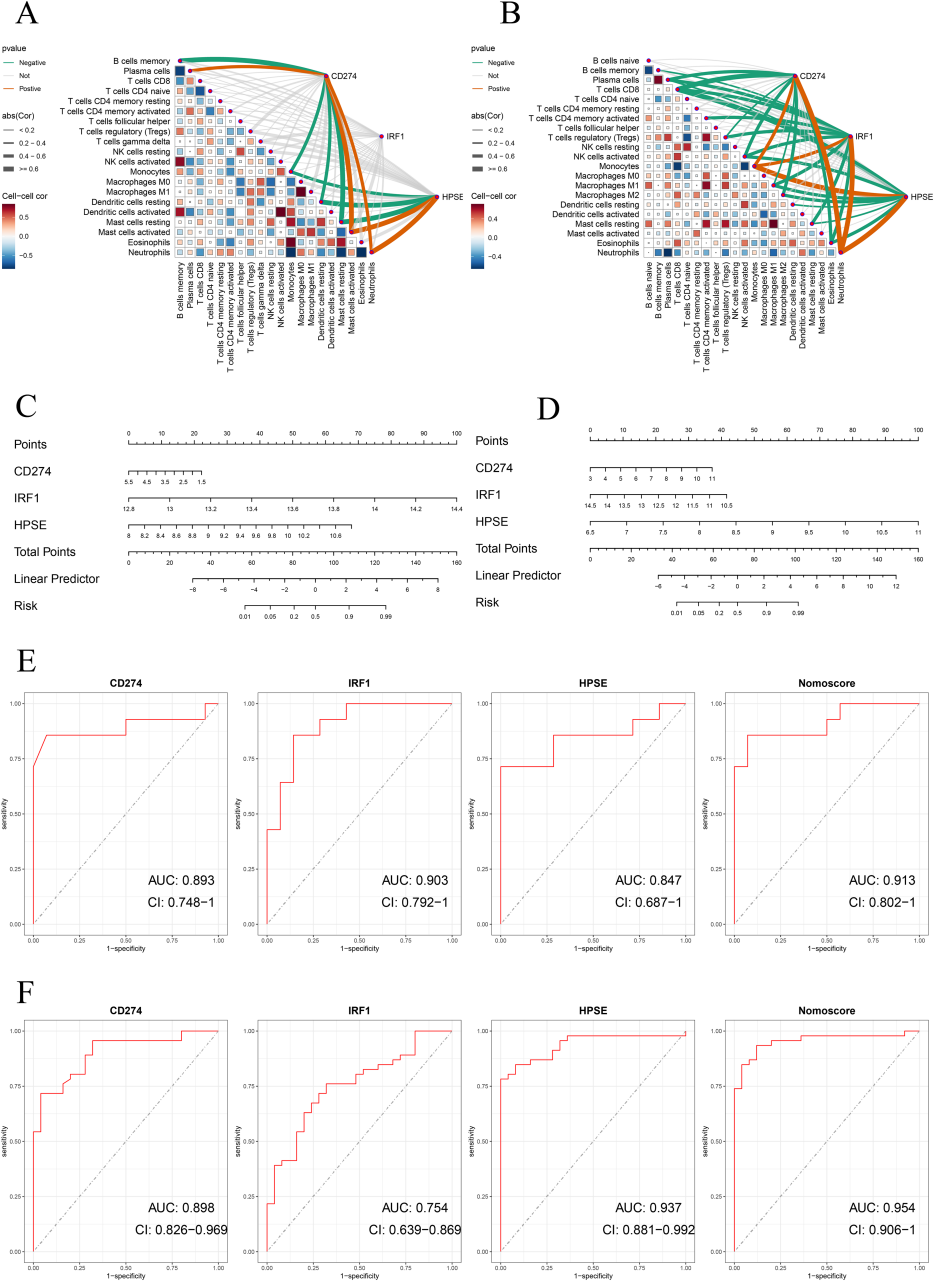
**(A, B)** Correlation analysis of neutrophil extracellular traps (NETs)-related hub genes with immune cell infiltration in validation datasets. Spearman’s rank correlation heatmaps show the association between expression levels of NETs-related hub genes (CD274, IRF1, HPSE) and immune cell subtypes in **(A)** GSE62525 and **(B)** GSE28623 datasets. Correlation coefficients are represented by color gradients (red: positive; blue: negative), and point sizes indicate statistical significance. Key immune cell subtypes include neutrophils, monocytes, CD8^+^ T cells, and NK cells. **(C, D)** Nomogram construction of three NETs-related hub genes in validation datasets for GSE62525 and GSE2862. **(E, F)** ROC curve of the three NETs-related hub genes and nomogram in validation datasets for GSE62525 and GSE2862.

In addition, to further evaluate the accuracy of the above results, this study conducted validation in two training datasets (GSE62525, GSE28623). Consistent with the previous results, in the training dataset GSE28623, CD274, IRF1, and HPSE were significantly positively correlated with neutrophils (**Figure 5B**). In the validation dataset GSE62525, CD274 and HPSE were significantly positively correlated with neutrophils. However, unfortunately, there was no significant correlation between IRF1 and neutrophils (**Figure 5A**). Moreover, the nomogram model and ROC curve analysis confirmed the good diagnostic value of the three genes (**Figures 5C–F**).

We further validated the diagnostic performance of the three hub genes (CD274, IRF1, HPSE) using precision-recall (PR) curves across all datasets (**Supplementary Figures 2**–**4**). The area under the PR curve (AUPR) for CD274, IRF1, and HPSE ranged from 0.875 to 0.971 in the cohorts (GSE62525: CD274 AUPR = 0.901, IRF1 AUPR = 0.859, HPSE AUPR = 0.896; GSE28623: CD274 AUPR = 0.859, IRF1 AUPR = 0.971, HPSE AUPR = 0.978; GSE19491: CD274 AUPR = 0.915, IRF1 AUPR = 0.875, HPSE AUPR = 0.948). The Nomoscore, integrating all three genes, achieved AUPR values exceeding 0.93 in all datasets (**Supplementary Figures 2**–**4**). These results corroborate the high diagnostic accuracy observed in ROC analyses. **Table 1** summarizes the specificity, sensitivity, F1 score, and 95% confidence intervals (CIs) for the hub genes across discovery and validation datasets.

**Table 1:** Diagnostic performance of NETs-related hub genes.

Dataset	Gene	Specificity	Sensitivity	F1 Score	AUC (95% CI)	AUPR
GSE19491	CD274	0.796296296	0.956521739	0.904109589	0.901 (0.84–0.962)	0.913
	IRF1	0.833333333	0.898550725	0.885714286	0.930 (0.886–0.975)	0.915
	HPSE	0.703703704	0.898550725	0.843537415	0.865 (0.798–0.933)	0.875
	Nomoscore	0.87037037	0.971014493	0.937062937	0.949 (0.908–0.99)	0.948
GSE62525	CD274	0.857142857	0.928571429	0.896551724	0.893(0.748−1)	0.933
	IRF1	0.857142857	0.857142857	0.857142857	0.903(0.792−1)	0.901
	HPSE	0.714285714	1	0.875	0.847(0.687−1)	0.896
	Nomoscore	0.857142857	0.928571429	0.896551724	0.913(0.802−1)	0.936
GSE28623	CD274	0.717391304	0.96	0.774193548	0.898(0.826−0.969)	0.948
	IRF1	0.760869565	0.68	0.641509434	0.754(0.639−0.869)	0.859
	HPSE	0.782608696	1	0.833333333	0.937(0.881−0.992)	0.971
	Nomoscore	0.934782609	0.88	0.88	0.954(0.906−1)	0.978

### Identification of drug-gene interactions

**Figure 6:**
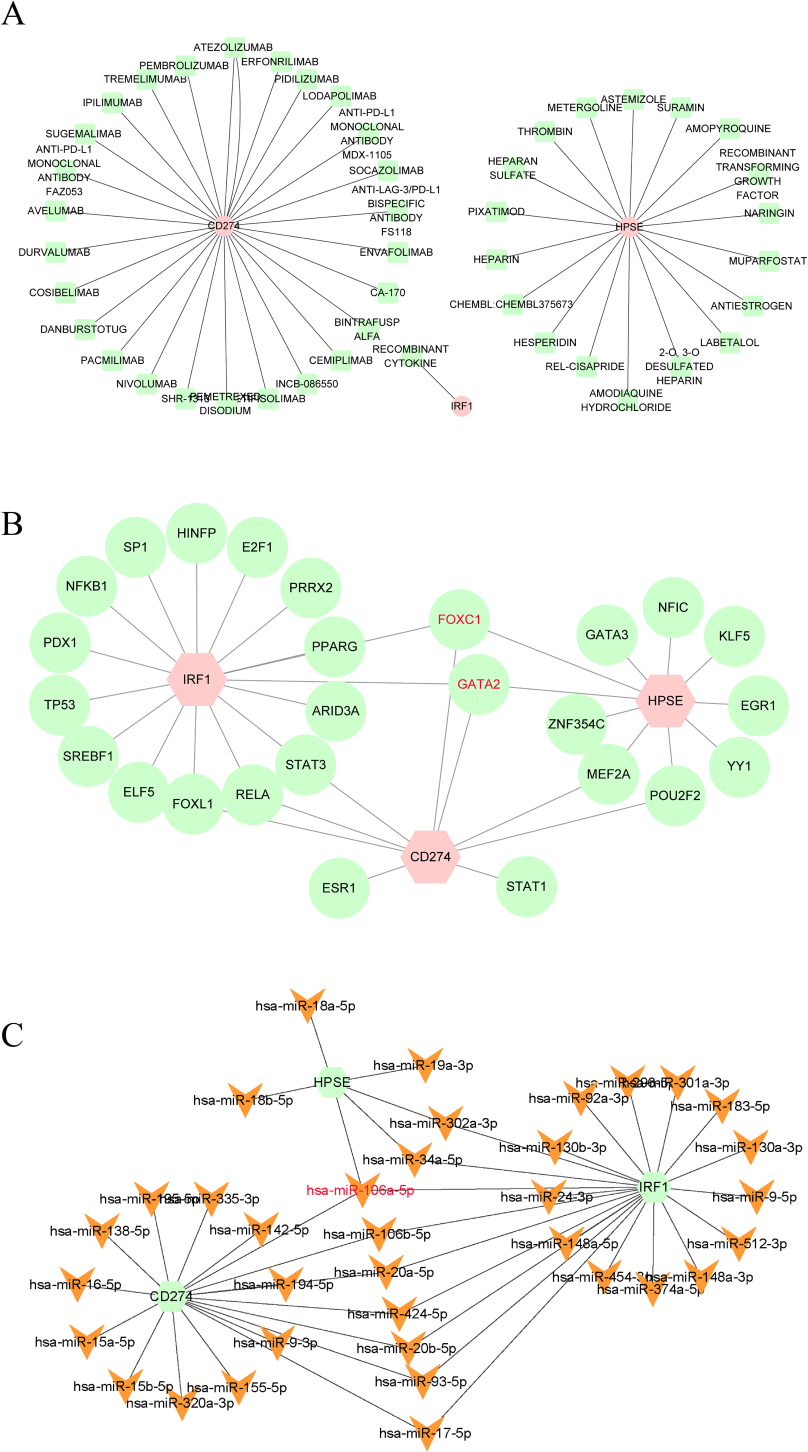
**(A)** Drugs-genes network of three hub genes. **(B)** TFs-genes regulatory network of three hub genes. **(C)** Genes-miRNAs regulatory network of three hub genes.

In this study, the DGIdb database was utilized to predict potential drugs that could interact with the hub genes related to NETs. A total of 46 potential drugs were screened out, including AMOPYROQUINE, RECOMBINANT CYTOKINE, PACMILIMAB, ENVAFOLIMAB, PIXATIMOD, and other drugs (**Supplementary File 4**). Additionally, in this study, Cytoscape 3.10.3 was employed to visualize the gene-drug interactions (**Figure 6A**).

### Construction of gene-TFs and gene-miRNAs interaction networks

In this study, we also constructed the gene-TFs and gene - miRNAs interaction networks to further explore the regulatory mechanisms of NETs-related hub genes. TFs and miRNAs have shown nonnegligible roles in the occurrence of diseases. Existing studies have demonstrated that TFs and miRNAs play crucial roles in the occurrence and development of TB (39–42). Therefore, in this study, we constructed the Gene-TFs and Gene-miRNAs interaction networks. According to the results (**Figures 6B, C**), FOXC1, GATA2, and hsa-miR-106a-5p interacted with the three hub genes, indicating that they may be the common regulatory factors of the three hub genes. FOXC1, GATA2, and hsa-miR-106a-5p may be the key core regulatory factors for the expression of the three hub genes.

## Discussion

TB remains a major global public health challenge. ATB is the most infectious form of TB, and the early detection of ATB and LTBI is crucial for the control and cure of TB. The IGRA and the TST are currently the most commonly used methods for TB diagnosis. However, unfortunately, neither of these two methods can distinguish between ATB and LTBI (13, 14). This diagnostic gap urgently requires the identification of reliable biomarkers that can accurately distinguish between these two states. In this study, we aimed to identify NETs- related biomarkers for ATB and LTBI through bioinformatics analysis and machine - learning algorithms. The identified hub genes, CD274, IRF1, and HPSE, exhibited significant diagnostic performance, which provides a potential breakthrough for the diagnosis of TB.

Neutrophils are a key cell type in the host immune response to TB, and their role in TB has gradually received increasing attention. They have both protective effects and potential hazards (15, 43–47). In this study, we identified 88 DE - NRGs associated with ATB. Through machine learning algorithms, including the least absolute shrinkage and LASSO, RF, and SVM-RFE, CD274, IRF1, and HPSE were screened as key hub genes. These genes exhibited powerful diagnostic performance in differentiating ATB from LTBI, and the ROCcurve analysis confirmed their high accuracy.

CD274, also known as programmed death - ligand 1 (PD - L1), is involved in immune regulation. Previous studies have shown (48) that it plays a crucial role in immune regulation and immune escape mechanisms of various diseases. In the context of TB, previous studies (49, 50) have demonstrated its involvement in immune regulation and its potential role in host pathogen interactions. Research has shown (49) that MTB infection can induce high expression of CD274 in macrophages and neutrophils, thereby weakening the host’s anti - TB immune response. This phenomenon is closely related to the activation of the NF-κB signaling pathway, which drives the release of inflammatory factors (such as TNF-α, IL-6) in TB and simultaneously promotes the expression of CD274 (50). Additionally, The upregulation of CD274 may contribute to the progression from latent infection to ATB by inhibiting T-cell proliferation (49). Therefore, CD274 not only serves as a diagnostic biomarker, but the immune checkpoint pathway it regulates may also provide a new target for TB immunotherapy. In our study, the expression of CD274 in ATB was significantly higher than that in LTBI (**Supplementary Figure 1**), with an AUC value of 0.901 in the training set, further emphasizing its diagnostic value. The upregulation of CD274 in ATB may reflect an adaptive immune escape strategy adopted by MTB. By interacting with receptors on immune cells, CD274 may inhibit the immune response and promote the survival and reproduction of pathogens. The high diagnostic accuracy of CD274, as well as its biological significance, make it a promising therapeutic target and object for diagnostic development.

IRF1, a key transcription factor in immune responses (51, 52). regulates genes critical for host defense against MTB (53). Moreover, IRF1 is involved in the regulation of the Th1-type immune response, and IFN-γ secreted by Th1 cells is a key factor in controlling MTB infection (52). However, overactivation of IRF1 may trigger an excessive inflammatory response, leading to lung tissue damage, a phenomenon particularly prominent in the pathological process of ATB patients (53). IRF1 showed significant differential expression between ATB and LTBI (**Supplementary Figure 1**). The high diagnostic accuracy of IRF1 (AUC: 0.93) highlights its potential as a biomarker. Notably, the expression of IRF1 was positively correlated with the abundance of neutrophils, further supporting its role in NET formation and immune regulation. Dysregulated IRF1 activity may lead to the excessive inflammatory response observed in ATB, exacerbating tissue damage and disease severity. Targeting the IRF1-related pathway may thus emerge as a novel therapeutic strategy to mitigate TB-related inflammation.

Among mammalian endoglycosidases, heparanase (HPSE) is currently the only known enzyme capable of cleaving heparan sulfate (HS). By cleaving heparan sulfate, it can regulate the remodeling process of the basement membrane and the extracellular matrix. Additionally, it can also promote the release of numerous HS-related molecules, including cytokines, growth factors, and various enzymes. In previous studies (54–58), HPSE has been shown to contribute to the occurrence, metastasis, drug resistance, and poor prognosis of various tumors. Some studies have indicated (54, 59) that HPSE may act as an effector component of NETosis and be released by neutrophils, leading to tissue damage. Additionally, HPSE exacerbates the inflammatory response by activating the TLR4/NF-κB pathway (62), which is associated with the formation of chronic granulomas in TB. This study shows that HPSE is significantly positively correlated with Neutrophils, with an AUC value of 0.865, indicating its diagnostic relevance.

The diagnostic potential of CD274, IRF1 and HPSE in active tuberculosis (ATB) is supported by their strong positive correlation with neutrophil infiltration, as shown in validation datasets (GSE62525 and GSE28623). These genes maintained their diagnostic accuracy in the validation datasets, consistent with the discovery dataset. However, the lack of a significant correlation between IRF1 and neutrophils in GSE62525 indicates potential dataset - specific differences and suggests that further validation in larger cohorts is necessary. While the observed correlations highlight the clinical relevance of these genes, the observational nature of transcriptomic data limits causal inference. Emerging mechanistic studies in non-TB models provide plausible hypotheses: CD274 (PD-L1) promotes NET release via PI3K/Akt/mTOR signaling in endotoxin-induced lung injury (60), IRF1 drives ROS-dependent NETosis in LPS-challenged neutrophils (61), HPSE facilitates NET extrusion through heparan sulfate cleavage in cancer-associated inflammation (54, 59). Notably, whether these pathways operate in MTB-infected neutrophils remains unproven. The dataset-specific discrepancy in IRF1-neutrophil correlations further underscores the need for functional validation in TB-specific contexts. Future studies should integrate neutrophil-specific gene perturbation (e.g., CRISPR/Cas9 knockout in primary human neutrophils infected with virulent MTB) with single-cell transcriptomics to resolve whether these hub genes are selectively expressed in NETosis-committed subsets. Such approaches will clarify if these genes act as drivers of NETosis or merely bystanders marking neutrophil activation, ultimately bridging the gap between correlation and causality in ATB pathogenesis.

To ensure the robustness of the research results, we validated the excellent diagnostic performance of CD274, IRF1, and HPSE in the validation datasets (GSE62525 and GSE28623). Consistent with the discovery dataset, these hub genes showed a significant positive correlation with neutrophil abundance in the validation datasets and maintained their diagnostic accuracy. However, IRF1 did not show a significant correlation with neutrophils in the GSE62525 dataset, indicating that there may be dataset - specific differences and further validation in a larger cohort is needed.

In this study, 46 candidate drugs with potential interactions with NETs-related hub genes (CD274, IRF1, HPSE) were screened out through the DGIdb database. Existing evidence shows that Iron dysregulation plays an important role in the pathogenesis of TB: hepcidin serum can significantly increase the susceptibility to TB (62), and the abnormally elevated serum hepcidin levels in patients coinfected with MTB and HIV are closely related to disease progression (63, 64). Heparin, as a hepcidin inhibitor, has been confirmed by research (65) to be able to significantly inhibit the expression of hepcidin in human macrophages after MTB infection, thereby effectively inhibiting the replication process of intracellular MTB. Suramin, as a drug for the treatment of trypanosomiasis, is considered to enhance the sensitivity of multidrug-resistant (MDR-TB) and extensively drug-resistant (XDR-TB) strains to existing antibiotics by inhibiting the SOS repair system mediated by the RecA protein of MTB (66). Recombinant human interleukin-2 (IL-2) (67) and interferon-gamma (IFN-γ) (68), as recombinant cytokines, have shown positive effects in the treatment of TB. In the list of candidate drugs in this study, there are also various monoclonal antibodies against programmed death receptor 1 (PD-1), such as Pembrolizumab, Nivolumab, etc. Although these drugs are currently mainly used in the treatment of cancer, numerous studies (69–74) have shown that anti-PD-1 therapy has also demonstrated encouraging positive effects in the treatment of TB. These findings suggest hypothetical therapeutic potential of candidate drugs, including heparin and PD-1 inhibitors, for further investigation in tuberculosis, although their clinical application necessitates rigorous risk-benefit analysis. For instance, the anticoagulant properties of heparin may significantly elevate bleeding risk in TB patients with comorbidities (e.g., cirrhosis or peptic ulcers), and this risk could be synergistically amplified by first-line anti-TB agents such as rifampicin and isoniazid, which are known to induce thrombocytopenia (75–77). Additionally, the immunosuppressive effects of PD-1 inhibitors might compromise host defense mechanisms against MTB potentially leading to MTB reactivation or secondary infections (78). Therefore, the hypothetical anti-TB effects of these candidates would require rigorous validation in preclinical models to assess efficacy and safety, followed by clinical trials to systematically assess safety profiles, with particular emphasis on risk stratification for bleeding complications and immunosuppression-related adverse events.

We constructed regulatory networks linking genes, transcription factors (TFs), and miRNAs, identifying FOXC1, GATA2, and hsa-miR-106a-5p as core regulators (79). FOXC1 and GATA2 are transcription factors that play crucial roles in cell differentiation and immune response (80). Regulation of these genes may alter immune response-related gene expression against MTB. As a microRNA, hsa-miR-106a-5p may regulate the expression of key genes at the post-transcriptional level. Understanding these regulatory mechanisms can provide a deeper insight into the molecular basis of ATB and contribute to the development of novel therapeutic strategies targeting these regulators.

In conclusion, as a key factor in the pathogenesis of TB, especially ATB, the study of NETs can not only provide new biomarkers for the early diagnosis of TB but also offer a new direction for immunotherapy. However, there are still some limitations. The three GEO datasets utilized in this study (GSE19491, GSE62525, GSE28623) exhibit inherent variability in sample size (e.g., ATB sample sizes: 54, 14, and 49, respectively) and population sources (e.g., geographic and clinical characteristics, as detailed in **Table 2**), which may introduce demographic or clinical heterogeneity into the gene expression profiles. For instance, GSE62525 has a relatively small sample size, and the datasets likely derive from distinct patient cohorts. Nevertheless, despite these differences, the three hub genes (CD274, IRF1, HPSE) demonstrated consistent diagnostic performance across independent validation sets (AUC >0.75 for all genes). Prior studies have similarly shown that biomarker-based diagnostic models maintain robustness across heterogeneous populations and sample sizes (15, 16). This study is based on the bioinformatics analysis of existing datasets, and the results need to be further verified in a larger clinical cohort and through experimental studies. Future studies could further leverage cutting-edge spatial omics technologies to deepen our understanding of NETs-related gene dynamics in TB pathogenesis. For instance, whole transcriptome co-mapping at cellular resolution with spatial CITE-seq (81) could validate the spatial expression patterns of CD274, IRF1, and HPSE within granulomas or inflammatory niches, clarifying their roles in local immune modulation. Additionally, spatially resolved *in vivo* CRISPR screen sequencing via perturb-DBiT (82) would enable functional dissection of these hub genes in NETosis and bacterial containment, directly testing their causality in TB progression. Beyond infectious diseases, integrating multimodal tri-omics mapping (e.g., transcriptome-epigenome-proteome) (83) could unravel the spatial dynamics of these genes in neuroinflammation or brain development, potentially identifying conserved regulatory networks across pathologies. These approaches would bridge molecular signatures to tissue-scale pathophysiology, accelerating therapeutic discovery.

**Table 2:** Details regarding the 3 data sets, test platforms, numbers of samples and source documentation.

Data Set	Platforms	LTBI sample size	ATB sample size	References (PMID)
GSE19491 (15)	GPL6947	69	54	20725040
GSE62525 (16)	GPL16951	14	14	26818387
GSE28623 (84)	GPL4133	25	49	25895988

## Data Availability

The original contributions presented in the study are included in the article/**Supplementary Material**. Further inquiries can be directed to the corresponding authors.

## References

[1] W.H. Organization. Global tuberculosis report (2024). Available online at: https://www.who.int/teams/global-tuberculosis-programme/tb-reports/global-tuberculosis-report-2024 (Accessed June 10, 2025).

[2] Marks SM Taylor Z Qualls NL Shrestha-Kuwahara RJ Wilce MA Nguyen CH. Outcomes of contact investigations of infectious tuberculosis patients. Am J Respir Crit Care Med. (2000) 162:2033–8. doi: 10.1164/ajrccm.162.6.2004022 PMC5448278 11112109

[3] American Thoracic Society, MMWR. Targeted tuberculin testing and treatment of latent tuberculosis infection. In: Recommendations and reports: Morbidity and mortality weekly report. Recommendations and reports, (United States: American Thoracic Society) vol. 49. (2000). p. 1–51. 10881762

[4] Kanabalan RD Lee LJ Lee TY Chong PP Hassan L Ismail R. Human tuberculosis and Mycobacterium tuberculosis complex: A review on genetic diversity, pathogenesis and omics approaches in host biomarkers discovery. Microbiological Res. (2021) 246:126674. doi: 10.1016/j.micres.2020.126674 33549960

[5] Scriba TJ Coussens AK Fletcher HA. Human immunology of tuberculosis. Microbiol Spectr. (2016) 4:TBTB2-0016-2016. doi: 10.1128/microbiolspec.TBTB2-0016-2016 27726784

[6] Borkute RR Woelke S Pei G Dorhoi A. Neutrophils in tuberculosis: cell biology, cellular networking and multitasking in host defense. Int J Mol Sci. (2021) 22:4801. doi: 10.3390/ijms22094801 33946542 PMC8125784

[7] Hilda JN Das S Tripathy SP Hanna LE. Role of neutrophils in tuberculosis: A bird’s eye view. Innate Immun. (2020) 26:240–7. doi: 10.1177/1753425919881176 PMC7251797 31735099

[8] Panteleev AV Nikitina IY Burmistrova IA Kosmiadi GA Radaeva TV Amansahedov RB. Severe tuberculosis in humans correlates best with neutrophil abundance and lymphocyte deficiency and does not correlate with antigen-specific CD4 T-cell response. Front Immunol. (2017) 8:963. doi: 10.3389/fimmu.2017.00963 28871253 PMC5566990

[9] Parker HA Forrester L Kaldor CD Dickerhof N Hampton MB. Antimicrobial activity of neutrophils against mycobacteria. Front Immunol. (2021) 12:782495. doi: 10.3389/fimmu.2021.782495 35003097 PMC8732375

[10] Arcos J Diangelo LE Scordo JM Sasindran SJ Moliva JI Turner J. Lung mucosa lining fluid modification of mycobacterium tuberculosis to reprogram human neutrophil killing mechanisms. J Infect Dis. (2015) 212:948–58. doi: 10.1093/infdis/jiv146 PMC4548464 25748325

[11] Ramos-Kichik V Mondragón-Flores R Mondragón-Castelán M Gonzalez-Pozos S Muñiz-Hernandez S Rojas-Espinosa O. Neutrophil extracellular traps are induced by Mycobacterium tuberculosis. Tuberculosis (Edinburgh Scotland). (2009) 89:29–37. doi: 10.1016/j.tube.2008.09.009 19056316

[12] Van Der Meer AJ Zeerleder S Blok DC Kager LM Lede IO Rahman W. Neutrophil extracellular traps in patients with pulmonary tuberculosis. Respir Res. (2017) 18:181. doi: 10.1186/s12931-017-0663-1 29084563 PMC5663039

[13] Pai M Behr M. Latent mycobacterium tuberculosis infection and interferon-gamma release assays. Microbiol Spectr. (2016) 4:TBTB2-0023-2016. doi: 10.1128/microbiolspec.TBTB2-0023-2016 27763261

[14] Ludi Z Sule AA Samy RP Putera I Schrijver B Hutchinson PE. Diagnosis and biomarkers for ocular tuberculosis: From the present into the future. Theranostics. (2023) 13:2088–113. doi: 10.7150/thno.81488 PMC10157737 37153734

[15] Berry MP Graham CM Mcnab FW Xu Z Bloch SA Oni T. An interferon-inducible neutrophil-driven blood transcriptional signature in human tuberculosis. Nature. (2010) 466:973–7. doi: 10.1038/nature09247 PMC3492754 20725040

[16] Lee SW Wu LS Huang GM Huang KY Lee TY Weng JT. Gene expression profiling identifies candidate biomarkers for active and latent tuberculosis. BMC Bioinf. (2016) 17 Suppl 1:3. doi: 10.1186/s12859-015-0848-x PMC4895247 26818387

[17] Shanmugasundaram U Bucsan AN Ganatra SR Ibegbu C Quezada M Blair RV. Pulmonary Mycobacterium tuberculosis control associates with CXCR3- and CCR6-expressing antigen-specific Th1 and Th17 cell recruitment. JCI Insight. (2020) 5:e137858. doi: 10.1172/jci.insight.137858 32554933 PMC7453885

[18] Tait DR Hatherill M Van Der Meeren O Ginsberg AM Van Brakel E Salaun B. Final analysis of a trial of M72/AS01(E) vaccine to prevent tuberculosis. N Engl J Med. (2019) 381:2429–39. doi: 10.1056/NEJMoa1909953 31661198

[19] Eum SY Kong JH Hong MS Lee YJ Kim JH Hwang SH. Neutrophils are the predominant infected phagocytic cells in the airways of patients with active pulmonary TB. Chest. (2010) 137:122–8. doi: 10.1378/chest.09-0903 PMC2803122 19749004

[20] Mayer-Barber KD Barber DL. Innate and adaptive cellular immune responses to mycobacterium tuberculosis infection. Cold Spring Harbor Perspect Med. (2015) 5:a018424. doi: 10.1101/cshperspect.a018424 PMC4665043 26187873

[21] Ulrichs T Kosmiadi GA Jörg S Pradl L Titukhina M Mishenko V. Differential organization of the local immune response in patients with active cavitary tuberculosis or with nonprogressive tuberculoma. J Infect Dis. (2005) 192:89–97. doi: 10.1086/430621 15942898

[22] Ulrichs T Kaufmann SH. New insights into the function of granulomas in human tuberculosis. J Pathol. (2006) 208:261–9. doi: 10.1002/path.1906 16362982

[23] Davis S Meltzer PS. GEOquery: a bridge between the gene expression omnibus (GEO) and bioConductor. Bioinf (Oxford England). (2007) 23:1846–7. doi: 10.1093/bioinformatics/btm254 17496320

[24] Wu J Zhang F Zheng X Zhang J Cao P Sun Z. Identification of renal ischemia reperfusion injury subtypes and predictive strategies for delayed graft function and graft survival based on neutrophil extracellular trap-related genes. Front Immunol. (2022) 13:1047367. doi: 10.3389/fimmu.2022.1047367 36532016 PMC9752097

[25] Zhang Y Guo L Dai Q Shang B Xiao T Di X. A signature for pan-cancer prognosis based on neutrophil extracellular traps. J immunotherapy Cancer. (2022) 10:e004210. doi: 10.1136/jitc-2021-004210 PMC9189842 35688556

[26] Li C Gao Z Su B Xu G Lin X. Data analysis methods for defining biomarkers from omics data. Analytical Bioanalytical Chem. (2022) 414:235–50. doi: 10.1007/s00216-021-03813-7 34951658

[27] Yu G Wang LG Han Y He QY. clusterProfiler: an R package for comparing biological themes among gene clusters. Omics: J Integr Biol. (2012) 16:284–7. doi: 10.1089/omi.2011.0118 PMC3339379 22455463

[28] Newman AM Liu CL Green MR Gentles AJ Feng W Xu Y. Robust enumeration of cell subsets from tissue expression profiles. Nat Methods. (2015) 12:453–7. doi: 10.1038/nmeth.3337 PMC4739640 25822800

[29] Huang H. linkET: everything is linkable. In: R package version 0.0.3. San Francisco, California, USA: GitHub (2021). Available at: https://github.com/Hy4m/linkET. (Accessed June 10, 2025).

[30] Wickham H. ggplot2: elegant graphics for data analysis. New York, NY, USA: Springer-Verlag New York (2016).

[31] Li Z Qin Y Liu X Chen J Tang A Yan S. Identification of predictors for neurological outcome after cardiac arrest in peripheral blood mononuclear cells through integrated bioinformatics analysis and machine learning. Funct Integr Genomics. (2023) 23:83. doi: 10.1007/s10142-023-01016-0 36930329 PMC10023777

[32] Meyer D Dimitriadou E Hornik K Weingessel A Leisch F Chang CC. Misc functions of the department of statistics. Wien TU, (Austria: TU Wien) editor (2015). doi: 10.32614/CRAN.package.e1071.

[33] Friedman J Hastie T Tibshirani R. Regularization paths for generalized linear models via coordinate descent. J Stat software. (2010) 33:1–22. doi: 10.18637/jss.v033.i01 PMC2929880 20808728

[34] Liaw A Wiener MC. Classification and regression by randomForest. (United States: R Foundation for Statistical Computing) (2007).

[35] Dusa A. venn: draw venn diagrams. (United Kingdom: Comprehensive R Archive Network (CRAN)) (2024). doi: 10.32614/CRAN.package.venn.

[36] Cannon M Stevenson J Stahl K Basu R Coffman A Kiwala S. DGIdb 5.0: rebuilding the drug-gene interaction database for precision medicine and drug discovery platforms. Nucleic Acids Res. (2024) 52:D1227–d1235. doi: 10.1093/nar/gkad1040 37953380 PMC10767982

[37] Zhou G Soufan O Ewald J Hancock REW Basu N Xia J. NetworkAnalyst 3.0: a visual analytics platform for comprehensive gene expression profiling and meta-analysis. Nucleic Acids Res. (2019) 47:W234–w241. doi: 10.1093/nar/gkz240 30931480 PMC6602507

[38] Skoufos G Kakoulidis P Tastsoglou S Zacharopoulou E Kotsira V Miliotis M. TarBase-v9.0 extends experimentally supported miRNA–gene interactions to cell-types and virally encoded miRNAs. Nucleic Acids Res. (2023) 52:D304–10. doi: 10.1093/nar/gkad1071 PMC10767993 37986224

[39] Grant NL Kelly K Maiello P Abbott H O'connor S Lin PL. Mycobacterium tuberculosis-specific CD4 T cells expressing transcription factors T-bet or RORγT associate with bacterial control in granulomas. mBio. (2023) 14:e0047723. doi: 10.1128/mbio.00477-23 37039646 PMC10294621

[40] Yang T Ge B. miRNAs in immune responses to Mycobacterium tuberculosis infection. Cancer Lett. (2018) 431:22–30. doi: 10.1016/j.canlet.2018.05.028 29803788

[41] Sinigaglia A Peta E Riccetti S Venkateswaran S Manganelli R Barzon L. Tuberculosis-associated microRNAs: from pathogenesis to disease biomarkers. Cells. (2020) 9:2160. doi: 10.3390/cells9102160 32987746 PMC7598604

[42] Gong Z Li H Cai Y Stojkoska A Xie J. Biology of MarR family transcription factors and implications for targets of antibiotics against tuberculosis. J Cell Physiol. (2019) 234:19237–48. doi: 10.1002/jcp.28720 31012115

[43] Brinkmann V Reichard U Goosmann C Fauler B Uhlemann Y Weiss DS. Neutrophil extracellular traps kill bacteria. Sci (New York N.Y.). (2004) 303:1532–5. doi: 10.1126/science.1092385 15001782

[44] Papayannopoulos V. Neutrophil extracellular traps in immunity and disease, Nature reviews. Immunology. (2018) 18:134–47. doi: 10.1038/nri.2017.105 28990587

[45] Cavalcante-Silva LHA Almeida FS Andrade AG Comberlang FC Cardoso LL Vanderley SER. Mycobacterium tuberculosis in a trap: the role of neutrophil extracellular traps in tuberculosis. Int J Mol Sci. (2023) 24:11385. doi: 10.3390/ijms241411385 37511144 PMC10379580

[46] Filio-Rodríguez G Estrada-García I Arce-Paredes P Moreno-Altamirano MM Islas-Trujillo S Ponce-Regalado MD. *In vivo* induction of neutrophil extracellular traps by Mycobacterium tuberculosis in a Guinea pig model. Innate Immun. (2017) 23:625–37. doi: 10.1177/1753425917732406 28929912

[47] Nakamura K Nakayama H Sasaki S Takahashi K Iwabuchi K. Mycobacterium avium-intracellulare complex promote release of pro-inflammatory enzymes matrix metalloproteinases by inducing neutrophil extracellular trap formation. Sci Rep. (2022) 12:5181. doi: 10.1038/s41598-022-09017-y 35410994 PMC9001666

[48] Topalian SL Drake CG Pardoll DM. Targeting the PD-1/B7-H1(PD-L1) pathway to activate anti-tumor immunity. Curr Opin Immunol. (2012) 24:207–12. doi: 10.1016/j.coi.2011.12.009 PMC3319479 22236695

[49] Yu XW Zhang JA Xie JP. Progress in PD-1/PD-L1, PD-L2 signaling pathway and its role in host anti-tuberculosis immunity. Zhonghua jie he he hu xi za zhi = Zhonghua jiehe he huxi zazhi = Chin J tuberculosis Respir Dis. (2024) 47:485–9. doi: 10.3760/cma.j.cn112147-20230904-00133 38706074

[50] Yang Y Fu Y Sheng S Ji C Pu X Xu G. Screening for diagnostic targets in tuberculosis and study on its pathogenic mechanism based on mRNA sequencing technology and miRNA-mRNA-pathway regulatory network. Front Immunol. (2023) 14:1038647. doi: 10.3389/fimmu.2023.1038647 36793717 PMC9923233

[51] Taniguchi T Ogasawara K Takaoka A Tanaka N. IRF family of transcription factors as regulators of host defense. Annu Rev Immunol. (2001) 19:623–55. doi: 10.1146/annurev.immunol.19.1.623 11244049

[52] Taki S Sato T Ogasawara K Fukuda T Sato M Hida S. Multistage regulation of Th1-type immune responses by the transcription factor IRF-1. Immunity. (1997) 6:673–9. doi: 10.1016/s1074-7613(00)80443-4 9208840

[53] Wu L Cheng Q Wen Z Song Y Zhu Y Wang L. IRF1 as a potential biomarker in Mycobacterium tuberculosis infection. J Cell Mol Med. (2021) 25:7270–9. doi: 10.1111/jcmm.16756 PMC8335664 34213077

[54] Hammond E Khurana A Shridhar V Dredge K. The role of heparanase and sulfatases in the modification of heparan sulfate proteoglycans within the tumor microenvironment and opportunities for novel cancer therapeutics. Front Oncol. (2014) 4:195. doi: 10.3389/fonc.2014.00195 25105093 PMC4109498

[55] Zheng LD Tong QS Tang ST Du ZY Liu Y Jiang GS. Expression and clinical significance of heparanase in neuroblastoma. World J pediatrics: WJP. (2009) 5:206–10. doi: 10.1007/s12519-009-0039-9 19693465

[56] Rivara S Milazzo FM Giannini G. Heparanase: a rainbow pharmacological target associated to multiple pathologies including rare diseases. Future medicinal Chem. (2016) 8:647–80. doi: 10.4155/fmc-2016-0012 27057774

[57] Edovitsky E Elkin M Zcharia E Peretz T Vlodavsky I. Heparanase gene silencing, tumor invasiveness, angiogenesis, and metastasis. J Natl Cancer Institute. (2004) 96:1219–30. doi: 10.1093/jnci/djh230 15316057

[58] Shteingauz A Boyango I Naroditsky I Hammond E Gruber M Doweck I. Heparanase enhances tumor growth and chemoresistance by promoting autophagy. Cancer Res. (2015) 75:3946–57. doi: 10.1158/0008-5472.Can-15-0037 PMC4573896 26249176

[59] Ishai-Michaeli R Eldor A Vlodavsky I. Heparanase activity expressed by platelets, neutrophils, and lymphoma cells releases active fibroblast growth factor from extracellular matrix. Cell Regul. (1990) 1:833–42. doi: 10.1091/mbc.1.11.833 PMC362850 2088528

[60] Zhu CL Xie J Zhao ZZ Li P Liu Q Guo Y. PD-L1 maintains neutrophil extracellular traps release by inhibiting neutrophil autophagy in endotoxin-induced lung injury. Front Immunol. (2022) 13:949217. doi: 10.3389/fimmu.2022.949217 36016930 PMC9396256

[61] Liu S Yue Y Pan P Zhang L Su X Li H. IRF-1 intervention in the classical ROS-dependent release of NETs during LPS-induced acute lung injury in mice. Inflammation. (2019) 42:387–403. doi: 10.1007/s10753-018-0903-7 30315525

[62] Gangaidzo IT Moyo VM Mvundura E Aggrey G Murphree NL Khumalo H. Association of pulmonary tuberculosis with increased dietary iron. J Infect Dis. (2001) 184:936–9. doi: 10.1086/323203 11528590

[63] Minchella PA Armitage AE Darboe B Jallow MW Drakesmith H Jaye A. Elevated hepcidin at HIV diagnosis is associated with incident tuberculosis in a retrospective cohort study. Int J tuberculosis Lung disease: Off J Int Union against Tuberculosis Lung Dis. (2014) 18:1337–9. doi: 10.5588/ijtld.14.0143 25299867

[64] Kerkhoff AD Meintjes G Burton R Vogt M Wood R Lawn SD. Relationship between blood concentrations of hepcidin and anemia severity, mycobacterial burden, and mortality among patients with HIV-associated tuberculosis. J Infect Dis. (2016) 213:61–70. doi: 10.1093/infdis/jiv364 26136467 PMC4676545

[65] Abreu R Essler L Loy A Quinn F Giri P. Heparin inhibits intracellular Mycobacterium tuberculosis bacterial replication by reducing iron levels in human macrophages. Sci Rep. (2018) 8:7296. doi: 10.1038/s41598-018-25480-y 29740038 PMC5940867

[66] Zaragoza-Huesca D Rodenas MC Peñas-Martínez J Pardo-Sánchez I Peña-García J Espín S. Suramin, a drug for the treatment of trypanosomiasis, reduces the prothrombotic and metastatic phenotypes of colorectal cancer cells by inhibiting hepsin. BioMed Pharmacother. (2023) 168:115814. doi: 10.1016/j.biopha.2023.115814 37918256

[67] Zhang R Xi X Wang C Pan Y Ge C Zhang L. Therapeutic effects of recombinant human interleukin 2 as adjunctive immunotherapy against tuberculosis: A systematic review and meta-analysis. PloS One. (2018) 13:e0201025. doi: 10.1371/journal.pone.0201025 30024982 PMC6053227

[68] Reljic R. IFN-gamma therapy of tuberculosis and related infections. J Interferon Cytokine research: Off J Int Soc Interferon Cytokine Res. (2007) 27:353–64. doi: 10.1089/jir.2006.0103 17523867

[69] Kee S-J Kwon Y-S Park Y-W Cho Y-N Lee S-J Kim T-J. Dysfunction of natural killer T cells in patients with active Mycobacterium tuberculosis infection. Infect Immun. (2012) 80:2100–8. doi: 10.1128/iai.06018-11 PMC3370582 22409933

[70] Singh A Dey AB Mohan A Mitra DK. Programmed death-1 receptor suppresses γ-IFN producing NKT cells in human tuberculosis. Tuberculosis (Edinburgh Scotland). (2014) 94:197–206. doi: 10.1016/j.tube.2014.01.005 24629634

[71] Adekambi T Ibegbu CC Kalokhe AS Yu T Ray SM Rengarajan J. Distinct effector memory CD4+ T cell signatures in latent Mycobacterium tuberculosis infection, BCG vaccination and clinically resolved tuberculosis. PloS One. (2012) 7:e36046. doi: 10.1371/journal.pone.0036046 22545156 PMC3335801

[72] Hassan SS Akram M King EC Dockrell HM Cliff JM. PD-1 PD-L1 and PD-L2 gene expression on T-cells and natural killer cells declines in conjunction with a reduction in PD-1 protein during the intensive phase of tuberculosis treatment. PloS One. (2015) 10:e0137646. doi: 10.1371/journal.pone.0137646 26359860 PMC4567315

[73] Zumla A Rao M Parida SK Keshavjee S Cassell G Wallis R. Inflammation and tuberculosis: host-directed therapies. J Intern Med. (2015) 277:373–87. doi: 10.1111/joim.12256 24717092

[74] Bandaru A Devalraju KP Paidipally P Dhiman R Venkatasubramanian S Barnes PF. Phosphorylated STAT3 and PD-1 regulate IL-17 production and IL-23 receptor expression in Mycobacterium tuberculosis infection. Eur J Immunol. (2014) 44:2013–24. doi: 10.1002/eji.201343680 PMC4106993 24643836

[75] Martins MA Reis AM Sales MF Nobre V Ribeiro DD Rocha MO. Rifampicin-warfarin interaction leading to macroscopic hematuria: a case report and review of the literature. BMC Pharmacol Toxicol. (2013) 14:27. doi: 10.1186/2050-6511-14-27 23641931 PMC3653703

[76] Hwang KW Choi JH Lee SY Lee SH Chon MK Lee J. Oral anticoagulants and concurrent rifampin administration in tuberculosis patients with non-valvular atrial fibrillation. BMC Cardiovasc Disord. (2023) 23:182. doi: 10.1186/s12872-023-03212-z 37016321 PMC10074893

[77] Kuwabara G Tazoe K Imoto W Yamairi K Shibata W Oshima K. Isoniazid-induced immune thrombocytopenia. Intern Med. (2021) 60:3639–43. doi: 10.2169/internalmedicine.6520-20 PMC8666230 34053983

[78] Chai Q Lu Z Liu CH. Host defense mechanisms against Mycobacterium tuberculosis. Cell Mol Life Sci. (2020) 77:1859–78. doi: 10.1007/s00018-019-03353-5 PMC11104961 31720742

[79] Onodera K Fujiwara T Onishi Y Itoh-Nakadai A Okitsu Y Fukuhara N. GATA2 regulates dendritic cell differentiation. Blood. (2016) 128:508–18. doi: 10.1182/blood-2016-02-698118 PMC5026465 27259979

[80] Wang Y Ma X Huang J Yang X Kang M Sun X. Somatic FOXC1 insertion mutation remodels the immune microenvironment and promotes the progression of childhood acute lymphoblastic leukemia. Cell Death Dis. (2022) 13:431. doi: 10.1038/s41419-022-04873-y 35504885 PMC9065155

[81] Liu Y Distasio M Su G Asashima H Enninful A Qin X. High-plex protein and whole transcriptome co-mapping at cellular resolution with spatial CITE-seq. Nat Biotechnol. (2023) 41:1405–9. doi: 10.1038/s41587-023-01676-0 PMC10567548 36823353

[82] Baysoy A Tian X Zhang F Renauer P Bai Z Shi H. Spatially Resolved *in vivo* CRISPR Screen Sequencing via Perturb-DBiT. bioRxiv: preprint server Biol. (2024). doi: 10.1101/2024.11.18.624106

[83] Zhang D Rubio Rodríguez-Kirby LA Lin Y Song M Wang L Wang L. Spatial dynamics of mammalian brain development and neuroinflammation by multimodal tri-omics mapping. bioRxiv: preprint server Biol. (2024). doi: 10.1101/2024.07.28.605493

[84] Elliott TO Owolabi O Donkor S Kampmann B Hill PC Ottenhoff TH. Dysregulation of apoptosis is a risk factor for tuberculosis disease progression. J Infect Dis. (2015) 212:1469–79. doi: 10.1093/infdis/jiv238 25895988

