# Supplementary figures and images for: Identification and validation of NETs-related biomarkers in active tuberculosis through bioinformatics analysis and machine learning algorithms
Source: Front Immunol. 2025 Jun 18;16:1599667. doi: 10.3389/fimmu.2025.1599667 (PMC12213393; doi:10.3389/fimmu.2025.1599667)

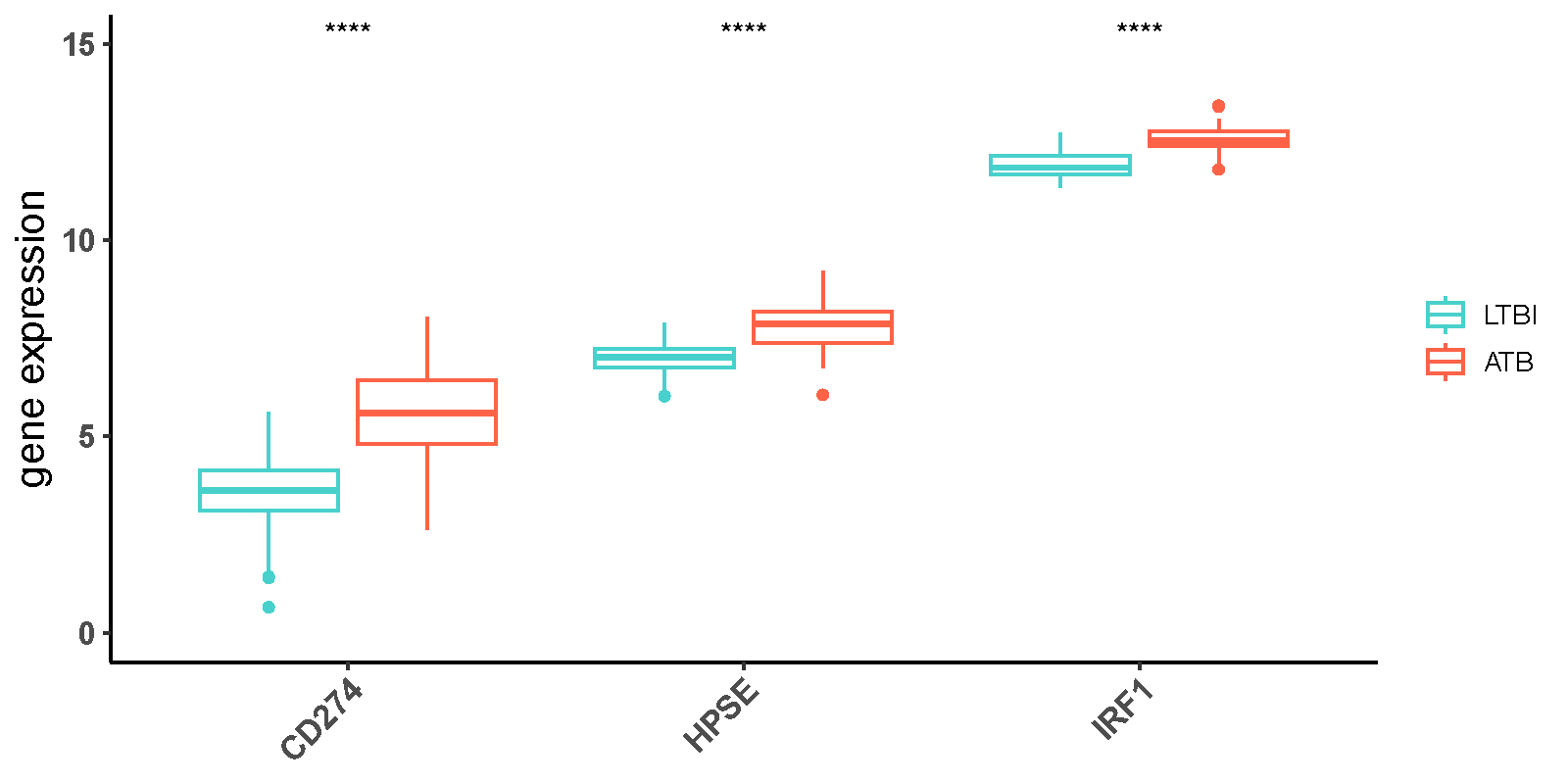

Supplement: Supplementary file 1 [file Image1.tiff]

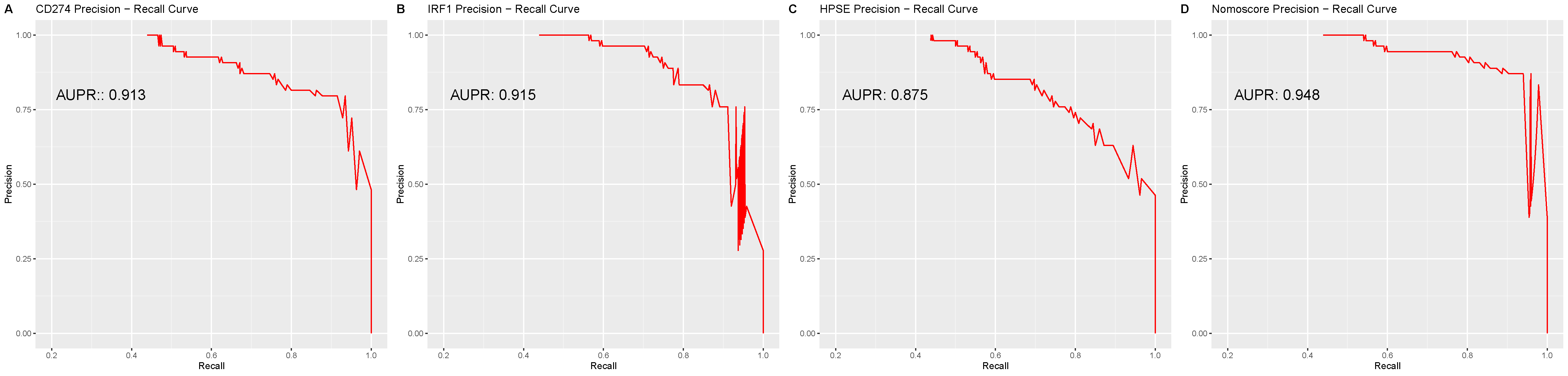

Supplement: Supplementary file 2 [file Image2.tiff]

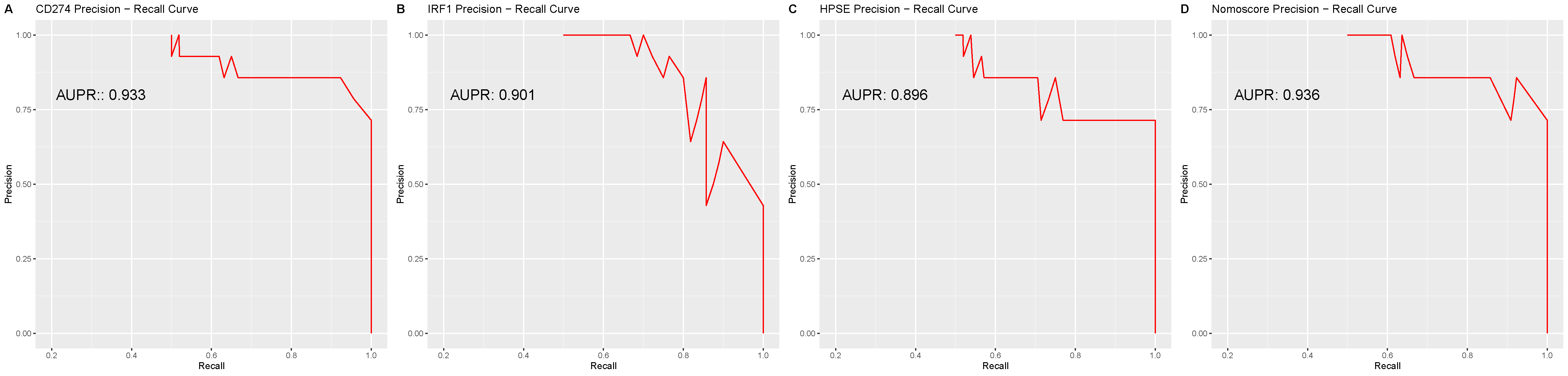

Supplement: Supplementary file 3 [file Image3.tiff]

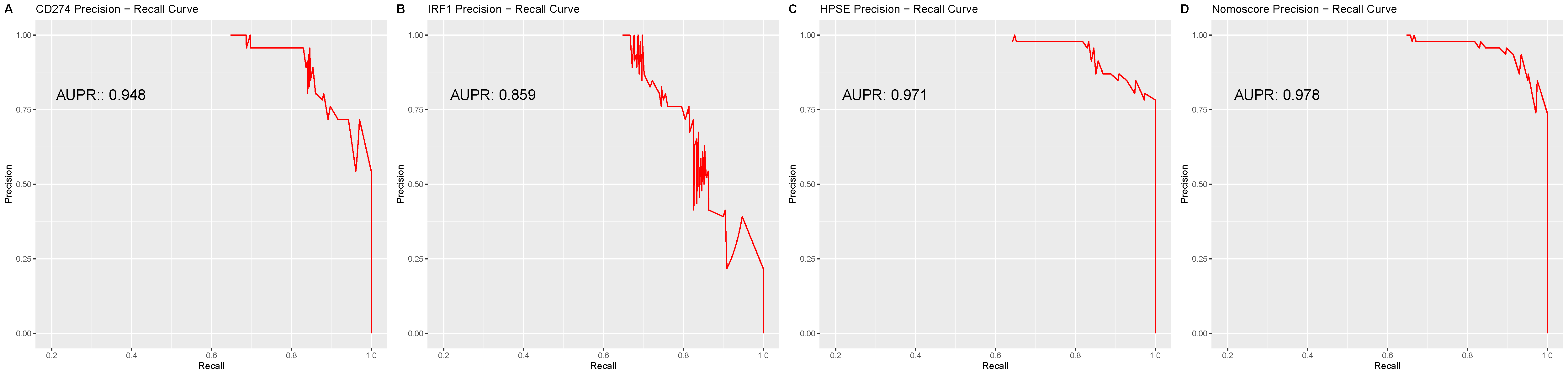

Supplement: Supplementary file 4 [file Image4.tiff]
